# Supplementary material for: A Strategy for the Production and Molecular Validation of Agrobacterium-Mediated Intragenic Octoploid Strawberry
Source: Plants (Basel). 2021 Oct 20;10(11):2229. doi: 10.3390/plants10112229 (PMC8622968; doi:10.3390/plants10112229)

Supplementary Table S1 List of primers used for vector construction and PCR analysis of transformants.

Primer name	Sequence (5'-3')	Amplicon size (bp)	purpose
CM2268<br>CM2269	gtacggtcagtcccttATGGCCGGAGGTTTCTCG<br>caggagagttgttgatCTACAACTGTGTAACAGGATC	1590+32	FvSTP8 CDS with adaptor (lowercase letters)
ZT067<br>ZT068	aagggactgaccgtacccggggatcgat<br>atcaacaactctcctggcgcac	11772	Reverse PCR for vector backbone 1
CM2328<br>CM2329	gttacacagttgtagATTTTGAAGAAGCGTCAGGGTTC<br>cgaattaattcggggCACAACAAGCACAAAACTTACA	401+30	FvSTP8 terminator with adaptor (lowercase letters)
CM2269<br>ZT069	caggagagttgttgatCTACAACTGTGTAACAGGATC<br>ccccgaattaattcggcgttaattcag	10748	Reverse PCR for vector backbone 2
CM2140<br>CM2340	gtgatcgattccgagcaaac (3' end of promoter)<br>AGCAAGATCTTGAGCTGCAG (mid FvSTP8)	528	Direct PCR identification of transformants
pMDC162-Kan1<br>pMDC162-Kan2	caatgtcataccacttgtccgc<br>ctcatgagtgaggccgatg	400	PCR analysis the potential integration of backbone DNA

Supplementary Table S2 Sequence information of the oligonucleotides used for droplet digital PCR (ddPCR) in this study.

**Target**	**Oligo Name**	**Sequence (5'-3')**	**Amplicon (bp)**	**Note**
Reference gene (glutathione S-transferase DHAR)	Ref-F	AGGCACATGTATGTGTTCTCTCA	178	Oligos used match one copy per genome: FxaC_26g17540
	Ref-R	GTACAGTTTTGGTGCCAAGC		
	Ref-Probe	FAM-CAGTGACTTTCTCTCCAGCAATGT		
T-DNA region of pMF-P03606:FvSTP8	Intragene-F	GTGATCGATTCCGAGCAAAC	147	Oligos match the junction region of P03606 (intragene-F) and FvSTP8 CDS (Intragene-R) on the intragenic construct.
	Intragene-R	GAGAAACCTCCGGCCATAAG		
	Intragene-Probe	VIC-CGAATTCGACCCAGCTTTCTTGTA		

Figure S1. One-dimensional plot of droplets measured for fluorescence signal emitted from the endogenous reference gene *FaDHA*R (FAM™ labeled, positive droplets are blue) or the intragene FvKNOX Pro:*FvSTP8* (VIC™ labeled; positive droplets are red) in four distinct transformants (#) and untransformed cv. Shanghai Angel (SQ). Negative droplets are black.

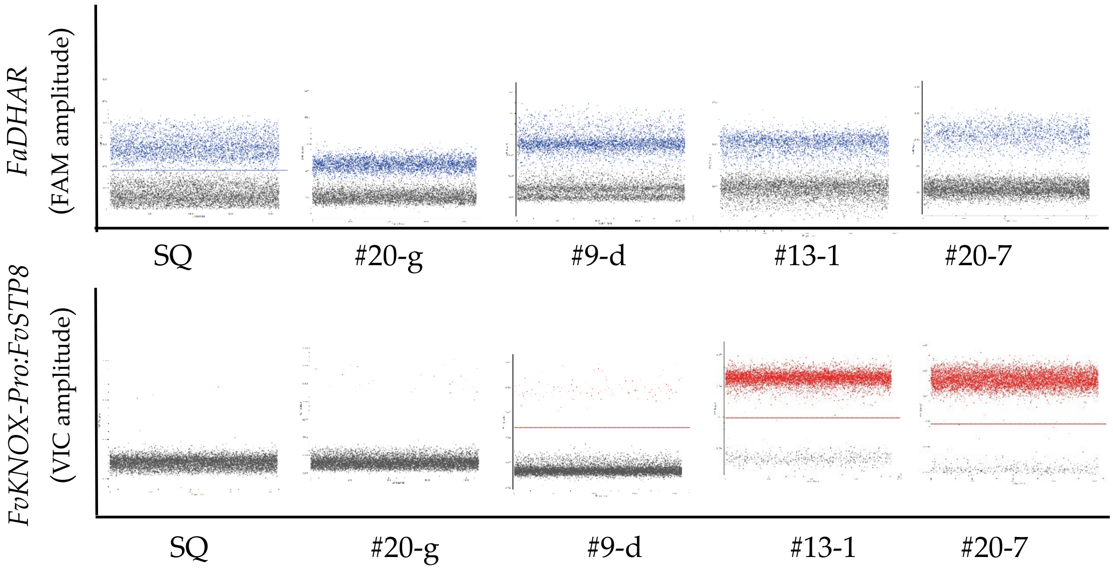

Figure S2. The droplets visualized in two dimensions for ddPCR analysis with the genomic DNA templates of SQ (untransformed Shanghai Angel), #20-g, #9-d and #20-7. The colors are as described in Fig.S1 but the droplets with both fluorescent probes are purple.

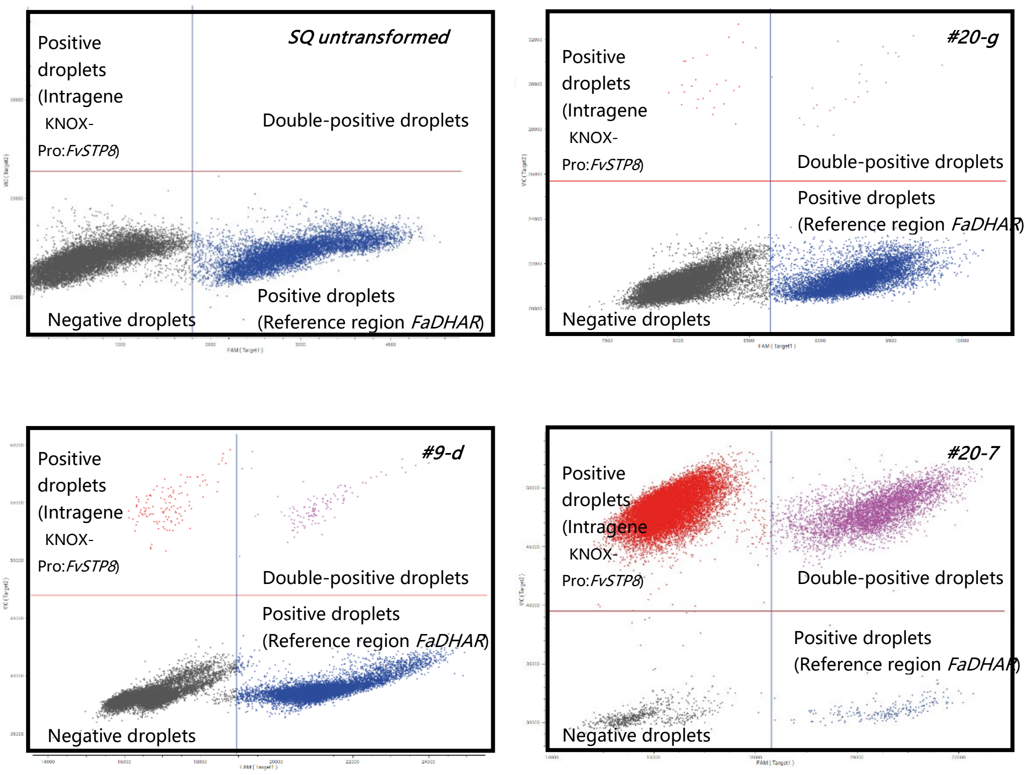

Supplement: Supplementary file 1 [file plants-10-02229-s001.zip › plants-1384732-supplementary.pdf]
